# An Abp1-Dependent Route of Endocytosis Functions when the Classical Endocytic Pathway in Yeast Is Inhibited

**DOI:** 10.1371/journal.pone.0103311

**Published:** 2014-07-29

**Authors:** Soheil Aghamohammadzadeh, Iwona I. Smaczynska-de Rooij, Kathryn R. Ayscough

**Affiliations:** Department of Biomedical Science, University of Sheffield, Sheffield, United Kingdom; Institute of Biology Valrose, France

## Abstract

Clathrin-mediated endocytosis (CME) is a well characterized pathway in both yeast and mammalian cells. An increasing number of alternative endocytic pathways have now been described in mammalian cells that can be both clathrin, actin, and Arf6- dependent or independent. In yeast, a single clathrin-mediated pathway has been characterized in detail. However, disruption of this pathway in many mutant strains indicates that other uptake pathways might exist, at least for bulk lipid and fluid internalization. Using a combination of genetics and live cell imaging, here we show evidence for a novel endocytic pathway in *S. cerevisiae* that does not involve several of the proteins previously shown to be associated with the ‘classic’ pathway of endocytosis. This alternative pathway functions in the presence of low levels of the actin-disrupting drug latrunculin-A which inhibits movement of the proteins Sla1, Sla2, and Sac6, and is independent of dynamin function. We reveal that in the absence of the ‘classic’ pathway, the actin binding protein Abp1 is now essential for bulk endocytosis. This novel pathway appears to be distinct from another described alternative endocytic route in *S. cerevisiae* as it involves at least some proteins known to be associated with cortical actin patches rather than being mediated at formin-dependent endocytic sites. These data indicate that cells have the capacity to use overlapping sets of components to facilitate endocytosis under a range of conditions.

## Introduction

Endocytosis is an essential eukaryotic cell process that is required to regulate cell surface composition [1]. In addition to this role, endocytic pathways are associated with a range of diseases including Alzheimers, Huntington's and cancer [2], [3], [4], [5]. They can also be exploited to serve as entry routes for pathogens and toxins, furthering the need to understand more about the complex mechanisms involved [6], [7].

Clathrin-mediated endocytosis (CME) is a well-characterized pathway in both yeast and mammalian cells. Several alternative endocytic pathways have now been described in mammalian cells that can be clathrin, actin, dynamin, Cdc42 and Arf6- dependent or independent [1]. The factors that determine the type of endocytic pathway used are still poorly understood. Research in the model organism *Saccharomyces cerevisiae* has been central to our current understanding of the mechanism of membrane invagination at the onset of the endocytic process. Some 50 proteins have been demonstrated to co-localize at endocytic, actin-associated patches in *S.cerevisiae*. To date, a single clathrin-mediated pathway has been described and studied in detail [8], [9], [10], [11]. This pathway is characterized by the sequential assembly of coat proteins and adaptors such as clathrin and the YAP180 homologues, followed by recruitment of actin polymerization machinery which facilitates the invagination of the membrane. Vesicle scission is then achieved by function of the yeast dynamin homologue Vps1 and an amphiphysin heterodimer, Rvs161/Rvs167 [12], [13]. Intriguingly, deletion of several components which block function of actin polymerization machinery, including the type 1 myosins (Myo3, Myo5) or the Wasp homologue (Las17), inhibit invagination but do not appear to preclude uptake of bulk lipid or fluid measured through use of the FM4-64 or Lucifer yellow dyes.

This continued uptake of membrane and fluid phase markers when the known pathway is inhibited indicates the possibility of other endocytic pathways. Genetic evidence, indicates that there is overlap, or redundancy, among some of the known actin patch components. For example, Abp1 is an actin-binding protein that co-localizes to actin patches at the cell surface but its deletion has no clear defects on the behaviour of commonly used endocytic reporters [9], [14]. *Abp1* deletion however, is lethal when combined with deletions in any of three other genes, *sla1*Δ, *sla2*Δ, and *sac6*Δ [15]. The proteins encoded by these other three genes all have defined roles and effects in classical endocytosis. This result has been taken to mean that there are multiple proteins performing the same role within a single endocytic patch complex. However, a second interpretation, is that yeast has distinct but overlapping endocytic routes, with Abp1 having the potential to function in a different pathway from the other three proteins. As well as the genetic evidence mentioned above, other data have been reported in the literature which constitute additional evidence for further endocytic pathways. For example, in the absence of *sla1*, fluid phase endocytosis continues but the endocytic component Sla2 and actin patches no longer co-localize indicating distinct functions and endocytic routes [16]. Furthermore, in the absence of an actin-binding protein Ysc84 which localizes to endocytic sites, the Arf3-GAP Lsb5 becomes essential [17]. Intriguingly, Arf3 is the yeast homologue of mammalian Arf6, a component in a subset of mammalian endocytic uptake processes [1].

Additional evidence for different pathways also arises from localization studies. Some proteins that are considered to have endocytic function, such as Ysc84, Abp1, and Arf3, localize primarily to the bud, whilst others, such as Sla2 and Ent1, are found distributed more evenly in mother and bud [16], [17], [18] and our unpublished work). Endocytosis in the mother cell is possibly primarily concerned with removal of unwanted or damaged receptors and transporters, while much of the endocytosis in the bud may be concerned with recycling of trafficking components, and ensuring maintenance of secretion and polarity.

A clathrin-independent pathway has been reported to function in yeast and was identified in cells with defective endocytic uptake caused by deletion of multiple ENTH domain containing proteins [19]. This alternative pathway involves a very distinct set of proteins from those characterized previously including proteins of the cell wall integrity pathway in conjunction with formin Bni1.

The work presented here adds to the growing evidence of alternative endocytic routes in yeast and opens the possibility of furthering our understanding of how cells are able to regulate uptake of distinct cargoes through distinct endocytic pathways.

## Materials and Methods

### Materials

Unless stated otherwise, chemicals were obtained from Sigma-Aldrich (St Louis, Missouri). Media was from Melford Laboratories, Ipswich, Suffolk, UK (yeast extract, peptone, agar) or Sigma (minimal synthetic medium and amino acids).

### Yeast strains, plasmids and cell growth

Yeast strains used in this study are listed in Table 1. Plasmids used were, pKA88, GFP-Abp1 *URA3*, *CEN*; pKA10, *ABP1*, *CEN*, *LEU2*; pKA735, *abp1*Δ*SH3, LEU2, CEN*. The acidic Abp1 mutants (pDD0863, 0865, 0866) were a gift from David Drubin (UC, Berkeley). Yeast cells were cultured using rotary shaking at 30°C in liquid YPD medium (1% yeast extract, 2% Bacto-peptone, 2% glucose supplemented with 40 µg/ml adenine) or in synthetic medium (0.67% yeast nitrogen base, 2% glucose) with appropriate supplements. GFP-Snc1 (a gift from H.Pelham, Cambridge) was integrated into the genome of KAY302 at the *URA3* locus after linearizing with Stu1 [20]. Latrunculin-A (Life Technologies Inc.) was added to cells at the concentration indicated from a 50 mM stock in DMSO.

**Table 1:** Yeast strains used in this study.

KAY	Genotype	Origin
1734	*MATa, his3-Δ200, leu2-3/112, ura3-52, trp1-1, lys2-801, Sla1-mRFP::HIS3, Sla2-GFP::HIS3*	KA lab
733	*MATa, his3-Δ1, leu2Δ, ura3Δ, met15Δ, Sla1-GFP::HIS3*	Euroscarf
302	*MATalpha, his3-Δ200, leu2-3,112, ura3-52, trp1-1, lys2-801*	KA lab
446	*MATa, his3-Δ1, leu2Δ, ura3Δ, met15Δ*	Euroscarf
633	KAY302 + *GFP-Snc1::URA3*	KA lab
126	*MATa, his3-Δ200, leu2-3,112, ura3-52, abp1*Δ::*LEU2*	KA lab
486	*MATa, his3-Δ1, leu2Δ, ura3Δ, met15Δ, rvs167*Δ::*KanMx*	Euroscarf
593	*MATa, his3-Δ1, le2uΔ, ura3Δ, met15Δ, sac6*Δ*KanMx*	Euroscarf
300	*MATa, his3-Δ200, leu2-3,112, ura3-52, trp1-1, lys2-801, sla1*Δ::*URA3*	KA lab
1095	*MATa, his3-Δ200, leu2-3,112, ura3-52, lys2-801, vps1*Δ::*KanMx*	Euroscarf
1170	*MATa, his3-Δ200, leu2-3,112, ura3-52, trp1-1, lys2-801, lsb5::LEU2*	KA lab
376	*MATa, his3-Δ200, leu2-3,112, ura3-52, trp1-1, lys2-801, ark1*Δ::*HIS3*	DDY1407 Drubin
381	*MATa, his3-Δ200, leu2-3,112, ura3-52, trp1-1, lys2-801, prk1*Δ::*LEU2*	DDY1559 Drubin
808	*MATa, his3-Δ1, leu2Δ, ura3Δ, met15Δ, srv2*Δ::*KanMx*	Euroscarf
138	*MATa, his3-Δ200, leu2-3,112, ura3-52, ade2-101, lys2-801, sla2Δ::HIS3*	KA lab
389	*MATa, his3-Δ200, leu2-3,112, ura3-52, trp1-1*	KA lab
1515	*MATa, his3-Δ1, leu2Δ, ura3Δ, met15Δ, Abp1-GFP:HIS3*	DDY3057 Drubin
1516	*MATalpha, his3-Δ200, leu2-3,112, ura3-52, Abp1-RFP::HIS3*	DD3058 Drubin

Lucifer yellow assay for fluid phase endocytosis was based on the method described by Dulic et al., [21]. Cells were grown in liquid YPAD to reach O.D_600 nm_ 0.25 after which 1 ml was spun down at 3000 rpm for 3 minutes. The pellet was resuspended in 30 µl YPAD and 10 µl of 40 mg/ml Lucifer yellow (Fluka) was added to the suspension. The suspension was incubated in a shaking incubator at 30°C for 2–90 minutes. Cells were washed 3 times with 1 ml ice-cold succinate-azide buffer (50 mM succinate, 20 mM sodium azide, pH 5.0, which acts as an energy poison). The pellet was then resuspended in 10 µl of succinate-azide buffer and left on ice until ready to be viewed by fluorescence microscopy (λ_ex_:428 nm; λ_em_: 530 nm).

*Yeast Vacuole Staining with FM4-64* (N-(triethylammoniumpropyl)-4-(p-diethylaminophenylhexatrienyl) pyridium dibromide; Invitrogen) is a lipophilic styryl dye used as a vital stain to follow bulk membrane internalization and transport to the vacuole in yeast [22]. 1 ml of log phase culture (OD_600 nm_≈0.5) was spun down at 3000 rpm for 3 minutes and resuspended in 500 µl YPAD. 0.5 µl of 16 mM stock FM-64 in DMSO was then added to the suspension (final concentration 16 µM) and incubated in a shaking incubator at 30°C for 10 minutes. Cells were centrifuged at 700 *g* for 3 minutes, resuspended in 1 ml YPAD and incubated for 30–60 minutes. Cells were viewed using a fluorescence microscope as described below (λ_ex_≈510 nm; λ_em_: 750 nm). Alternatively, the derivative FM4-64FX was added to a final concentration of 20 µM.

### Visualization of GFP-Tagged Proteins *In Vivo*

Cells were grown to mid-log phase in liquid drop-out media and pipetted onto glass slides, covered with a coverslip and viewed with an epifluorescence microscope.

### Phalloidin Staining of Filamentous actin

To visualize filamentous actin using fluorescence microscopy 0.134 ml 37% formaldehyde (Calbiochem) was added to 1 ml of actively growing cells (mid-log phase) and incubated at room temperature for 1 hour. The suspension was centrifuged and washed twice with 1 ml PBS+1 mg/ml BSA+0.1% Tx-100, the pellet was then resuspended in 50 µl of the buffer and 5 µl Rhodamine phalloidin (Invitrogen) was added and the cells were incubated in the dark for 30 minutes at room temperature. The pellet was washed twice with an excess volume of PBS/BSA solution and resuspended in 200 µl PBS/BSA before visualizing cells.

### Microscopy

Epifluorescence microscopy was performed using an Olympus IX-81 inverted microscope with a Photometrics Cool Snap HQ2 cooled CCD camera, and Image ProPlus image capture software. Alternatively images were captured using a Deltavision microscope on an Olympus IX-81 inverted microscope with a Olympus IX-HLSH100 camera and SoftWoRX software. For double labelled cells 0.5 sec exposures were used for imaging each FM dye and for GFP, and 2 sec lapse was used in movies (60 exposures – total movie time −120 sec).

Images were exported as TIFF files and assembled using Adobe Photoshop CS2. Kymographs were assembled using ImageJ software. Statistical analysis of localization was performed using Graphpad Prism software.

## Results

### Endocytosis at distinct sites and with different proteins

**Figure 1:**
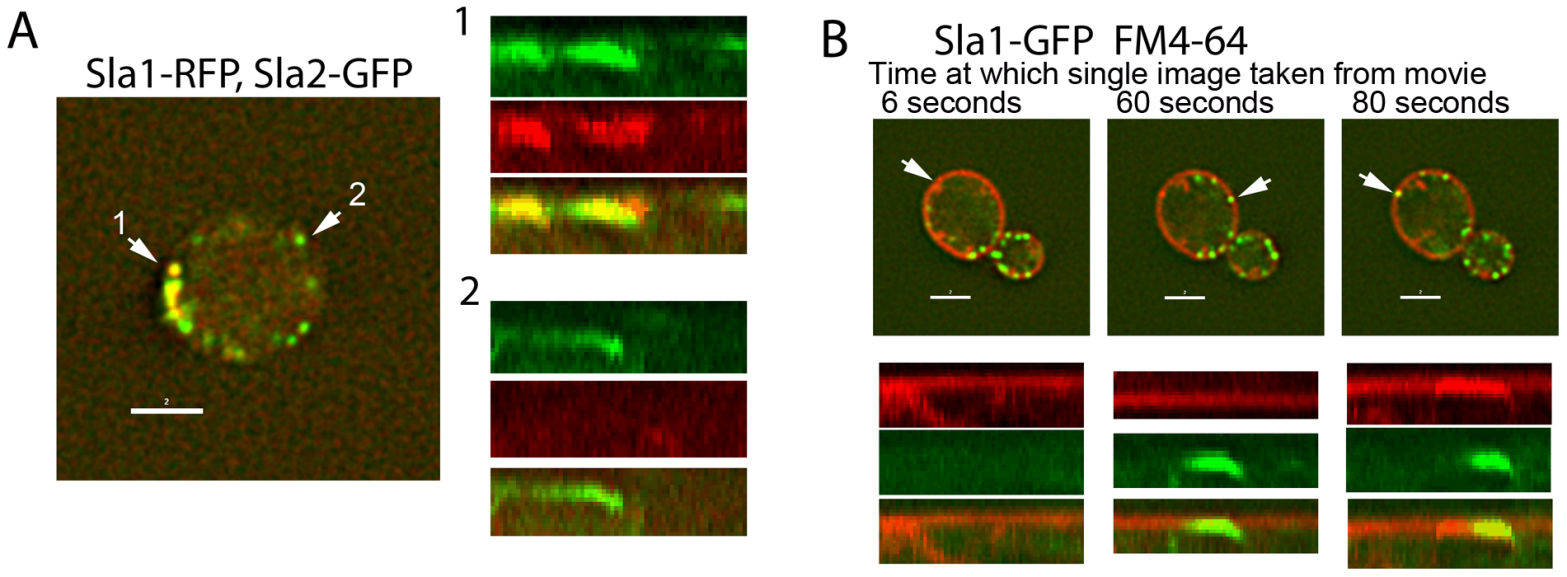
Alternative endocytic components and routes in wild type cells. (A) Cells co-expressing Sla1-mRFP and Sla2-GFP (KAY1734) were imaged and composition of spots was analysed. Kymographs show examples of puncta that show the two proteins together and with Sla2-GFP alone. Kymographs are from full movie (120 seconds) (B) Cells expressing Sla1-GFP (KAY733) were incubated with FM4-64FX for 5 minutes and imaged. Examples of both GFP only and FM4-64 only puncta invaginating are shown in kymographs. Kymographs are from full movie (120 seconds). The images are stills taken from supplemental movie at the times indicated. Scale bar = 2 µm.

While the classic model of yeast endocytosis suggests that all invaginations contain the same set of proteins required to drive the inward movement of the proteins, analysis of individual endocytic sites indicates the actual situation is more complex. For example, Sla1 is considered to be an endocytic adaptor that binds cargo and also facilitates association of the coat protein Sla2 to the actin polymerization machinery [16]. Analysis of cells expressing both Sla1-mRFP and Sla2-GFP demonstrates that while invagination of Sla1 and Sla2 does often occur at the same site, Sla2 patches does not absolutely require the presence of Sla1. This is demonstrated in the kymographs (Figure 1A) generated from time lapse movies (Movie S1). Thus, composition of endocytic sites can vary, and even in wild type cells not all recognized endocytic proteins need to be present for invagination to occur.

Further evidence for distinct endocytic routes can be gained by analysis of lipid using the dye FM4-64. If uptake of this dye is analysed in the presence of GFP-tagged Sla1 a subset of puncta (both Sla1-GFP and FM4-64) appear to enter the cell without showing any colocalization (kymographs, figure 1B; Movie S2). As shown in the figure, a single cells is capable of showing both co-localizing and non co-localizing patches. In cells incubated with FM4-64 for 5 minutes, analysis revealed that 60±9% of Sla1-GFP patches accumulated FM4-64. Strikingly however only 24±3% of FM4-64 puncta were associated with Sla1-GFP. The nature of the FM4-64 puncta was different from those associated with Sla1-GFP in that the puncta appeared more diffuse and were more dynamic having a lifetime of about 6 seconds compared to 20–30 seconds when co-localization was observed. These data indicate that some bulk uptake might occur using mechanisms additional to those operating in the usual route for endocytosis that is currently analysed by following specific fluorescently tagged reporter proteins in live cells.

### Inhibition of classical CME does not prevent fluid phase and bulk lipid endocytosis

**Figure 2:**
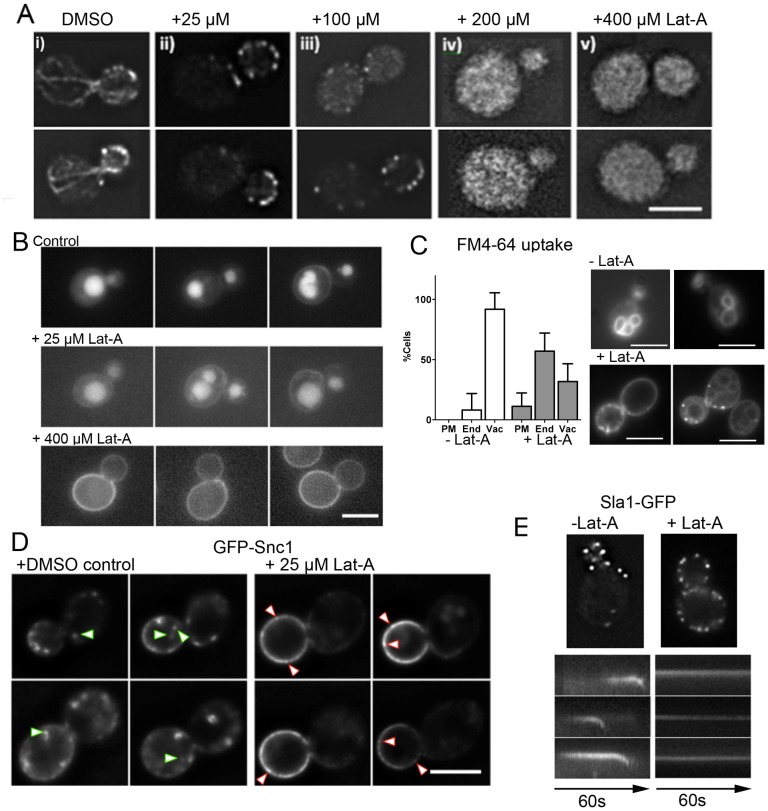
The Effect of Lat-A on actin and endocytosis. (A) Lat-A was added at the levels indicated for 15 minutes, before cells were fixed and labelled with rhodamine phalloidin to visualize F-actin. Bar = 5 µm. (B) The fluid phase dye Lucifer yellow was added to cells in the presence of 0, 25 or 400 µM Lat-A. Uptake of Lucifer yellow was assessed after 90 minutes. Bar = 5 µm. (C) Cells were treated with Lat-A 25 µM or DMSO (control) for 20 minutes before incubating with FM4-64. The localization of the dye was categorized as plasma membrane (PM), endosomal (End) or vacuolar (vac). Shown is the mean±Std Dev of 3 experiments. An unpaired students t-test indicates that there is a significant increase in endosomal staining in the treated cells p<0.0001. Examples of cells stained with FM4-64 in the absence or presence of Lat-A are shown. Bar = 5 µm (D) Strains expressing integrated GFP-Snc1 were imaged in the presence or absence of 25 µM Lat-A. Green arrowheads indicate internalising or internalized material. Red arrowheads show puncta of GFP-Snc1 at the plasma membrane. Bar = 5 µm. (E) Wild type cells expressing Sla1-GFP were grown to mid-log phase, half the sample was treated with Lat-A for 20 minutes. Time lapse movies were recorded over 90 seconds and kymographs generated.

While deletions and mutations in clathrin (heavy and light chains) and a number of other endocytic components including amphiphysins (Rvs161/Rvs167), Sla2/HIP1R and Sac6/fimbrin severely compromise endocytic uptake of cargoes including alpha factor receptor and GFP-Snc1 [23], [24], [25], [26], [27], uptake of FM4-64 often occurs relatively normally, albeit with some kinetic delay [22] and our unpublished observations). In previous work we demonstrated that addition of latrunculin-A (Lat-A), an actin monomer sequestering drug, disrupts actin organization and inhibits endocytosis [28]. Lat-A is routinely used at levels that result in the complete disassembly of cortical actin patches (200–400 µM). However further analysis of the effects of Lat-A has indicated that there are levels of this drug that do not appear to disrupt cortical actin patches and such levels can infact be remedial for cells with stabilized actin structures which themselves are linked with accumulation of reactive oxygen species [29]. To investigate the links between endocytosis and actin patches, wild type cells were grown to log phase and a range of levels of Lat-A (0, 25, 100, 200, 400 µM) were added for 10 minutes and the organization of F-actin analysed using rhodamine phalloidin staining. As shown, (figure 2A) at both 25 and 100 µM Lat-A, while actin cables appear to be disrupted, cortical actin sites remain intact, though the level of F-actin appears reduced in the cells incubated with 100 µM Lat-A. All actin structures were disrupted at levels of 200 and 400 µM Lat-A. The effect of 25 µM Lat-A on endocytosis was then determined. Four approaches were used; uptake of Lucifer yellow was used to determine any effects on fluid phase endocytosis; FM4-64 is a lipophylic dye used to determine membrane internalization; GFP-Snc1 is a reporter showing trafficking of a SNARE protein between the plasma membrane and endosomes; and analysis of Sla1-GFP allows the behaviour of individual endocytic sites to be assessed. As shown in figure 2B, in the presence of 25 µM Lat-A, Lucifer yellow was still internalized and trafficking to vacuoles was observed, while at 400 µM Lat-A uptake was completely abrogated. Analysis of lipid internalization using FM4-64 revealed that after 20 minutes incubation, 100% untreated cells internalized the dye and the majority of cells showed vacuolar staining. In the presence of 25 µM Lat-A 89±4% cells internalized the dye showing endocytosis was functioning, however there was a reduced number of cells with predominant vacuolar staining indicating a role for F-actin in post endosomal trafficking. A post-endosomal role for actin in yeast has previously been suggested [30]. These two approaches indicate that bulk endocytosis is not affected following addition of 25 µM Lat-A. GFP-Snc1 is a fluorescently tagged SNARE protein that has been used as a reporter for endosomal uptake and recycling [20], [23]. High throughput screens analysing uptake and recycling of the tagged SNARE GFP-Snc1 have indicated the importance of the recognized clathrin mediated endocytic pathway for its uptake into cells [26]. In wild type, untreated cells Snc1 is observed in puncta at the surface and also in discrete structures, presumed to be endosomes inside cells (Figure 2D). In the presence of 25 µM Lat-A, uptake is inhibited and localization is only seen at the cell surface. Inhibition of GFP-Snc1 uptake at levels of 25 µM Lat-A suggested that the CME pathway could be inhibited even when cortical patches are intact.

In order to address whether the known endocytic route was disrupted, the behaviour of a well characterized endocytic reporter protein Sla1-GFP was analysed further. Cells expressing Sla1-GFP were incubated in the presence of DMSO or 25 µM Lat-A, and after 20 minutes cells were imaged to analyse the behaviour of membrane associated Sla1-GFP patches (figure 2E). It was already known that Sla1 is able to localize to the plasma membrane at discrete sites in the absence of F-actin. However, it was unexpected that the low level of Lat-A would inhibit movement of this protein such that it was not able to invaginate. These data therefore demonstrate that 25 µM Lat-A does not disrupt cortical actin patch formation nor bulk lipid or fluid phase endocytosis. It does however prevent endocytosis of the cargo molecule Snc1 and movement of the endocytic component Sla1.

### A subset of known endocytic components are required for bulk lipid and fluid phase endocytosis in the presence of low levels of Lat-A

**Figure 3:**
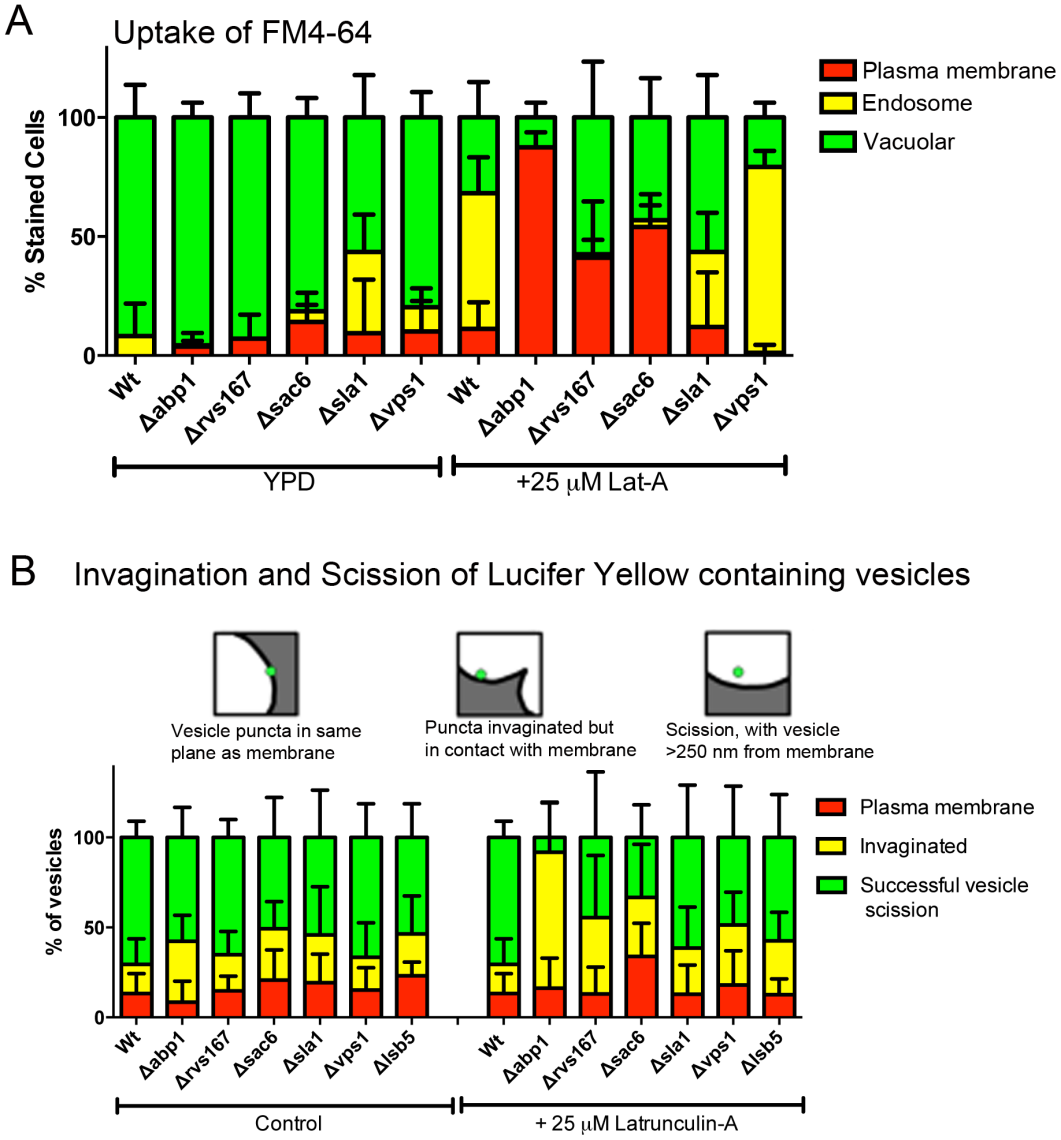
The Effect of Gene Deletions on Bulk Endocytosis in the presence and absence of 25 µM Lat-A. Different endocytic null mutant strains were grown to mid log phase and incubated with bulk endocytic markers in the absence of presence of 25 µM Lat-A. (A) FM4-64 uptake was assessed after 20 minutes incubation and categorized as being plasma membrane, endosomal or vacuolar. Error bars – std deviation. (B) Analysis of the status of LY puncta following incubation in the absence or presence of Lat-A. Categories determined (i) in the plane of the membrane, (ii) invaginated or (iii) successfully undergone scission. Number of vesicles counted ≥50 in ≥10 cells. Error is std deviation.

Given that the presence of 25 µM Lat-A does not block uptake of bulk lipid or fluid. We next asked whether deletion of known components of the endocytic machinery compromise this observed uptake route. Cells with deletions in a number of genes for endocytic components including the actin binding protein Abp1, the amphiphysin Rvs167, the actin bundling protein Sac6, the adaptor protein Sla1, and the dynamin homologue Vps1 were analysed for uptake of FM4-64 in the presence and absence of 25 µM Lat-A. As shown in figure 3A mutants showed small or no significant differences from wild type cells in uptake nor subsequent trafficking of the dye. In the presence of Lat-A three mutants, *abp1*Δ, *rvs167*Δ and *sac6*Δ showed an inhibition in initial internalization of the dye. Surprisingly, the strongest defect in endocytosis in these conditions was in the cells lacking *abp1* which, of the three mutations, causes the mildest effect on the classical endocytosis pathway.

The mutants were analysed further for uptake of the fluid phase dye Lucifer yellow. An additional deletion for *lsb5* (encoding an Arf3 GTPase Activating Protein; GAP) was included in this assay to indicate the possibility of Arf function in the pathway. The processing of cells following dye addition allows the stage of endocytosis when inhibition is occurring to be analysed in more detail. As before cells were grown to log phase and incubated with Lat-A for 20 minutes. Cells were stained with LY in the continued presence of Lat-A for a further 20 minutes before imaging as described. The LY patches at the plasma membrane were categorized as being in the plane of the membrane; invaginated but still associated with the membrane; or as having undergone scission where separation from the membrane is observed. As shown in figure3B, puncta in all three categories can be observed in DMSO treated cells with the majority in all cells being in the Scission category. In the presence of 25 µM Lat-A the same three mutants highlighted in the FM4-64 uptake experiment (*abp1*Δ, *rvs167*Δ and *sac6*Δ) all show a decrease in the puncta in the scission category with the strongest defect again being in the *abp1* null cells.

### The importance of Abp1 domains in this alternative endocytic route

**Figure 4:**
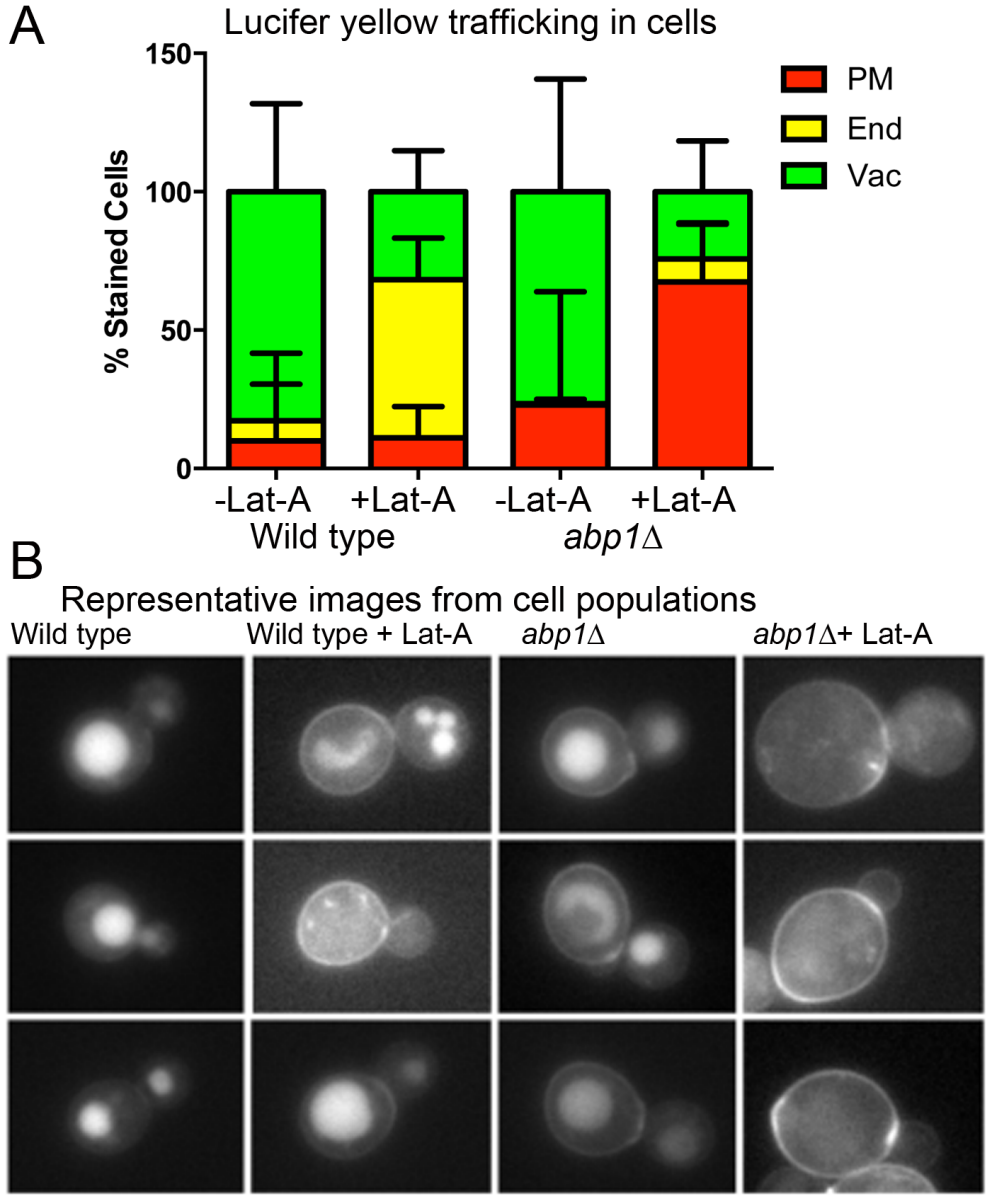
The effect of abp1 deletion on the uptake and trafficking of Lucifer yellow. (A) Wild type and *abp1*Δ cells were grown to mid log phase. Half of the cells were incubated with 25 µM Lat-A. LY was added for 90 minutes as described. Localization of stain was categorized as being at the plasma membrane, endosomes or vacuoles. Error bars are std deviation. (B) Representative images from the cells visualized. Bar = 5 µm.

The analysis above indicates that at relatively short time points (up to 20 minutes) after addition, Lucifer yellow accumulates at the plasma membrane in *abp1* null cells in the presence of low Lat-A levels. To determine whether this accumulation represents a kinetic delay or a more effective endocytic block the Lucifer yellow uptake was monitored for 90 minutes. As shown (figure 4A,B) after this prolonged incubation wild type cells treated with Lat-A and Lucifer yellow have a low level of membrane staining and also show an increase in cells with endosomal rather than just vacuolar staining. In *abp1* null cells treated with Lat-A 68±10% of cells continue to have puncta associated with the plasma membrane compared to <10% of wild type cells.

**Figure 5:**
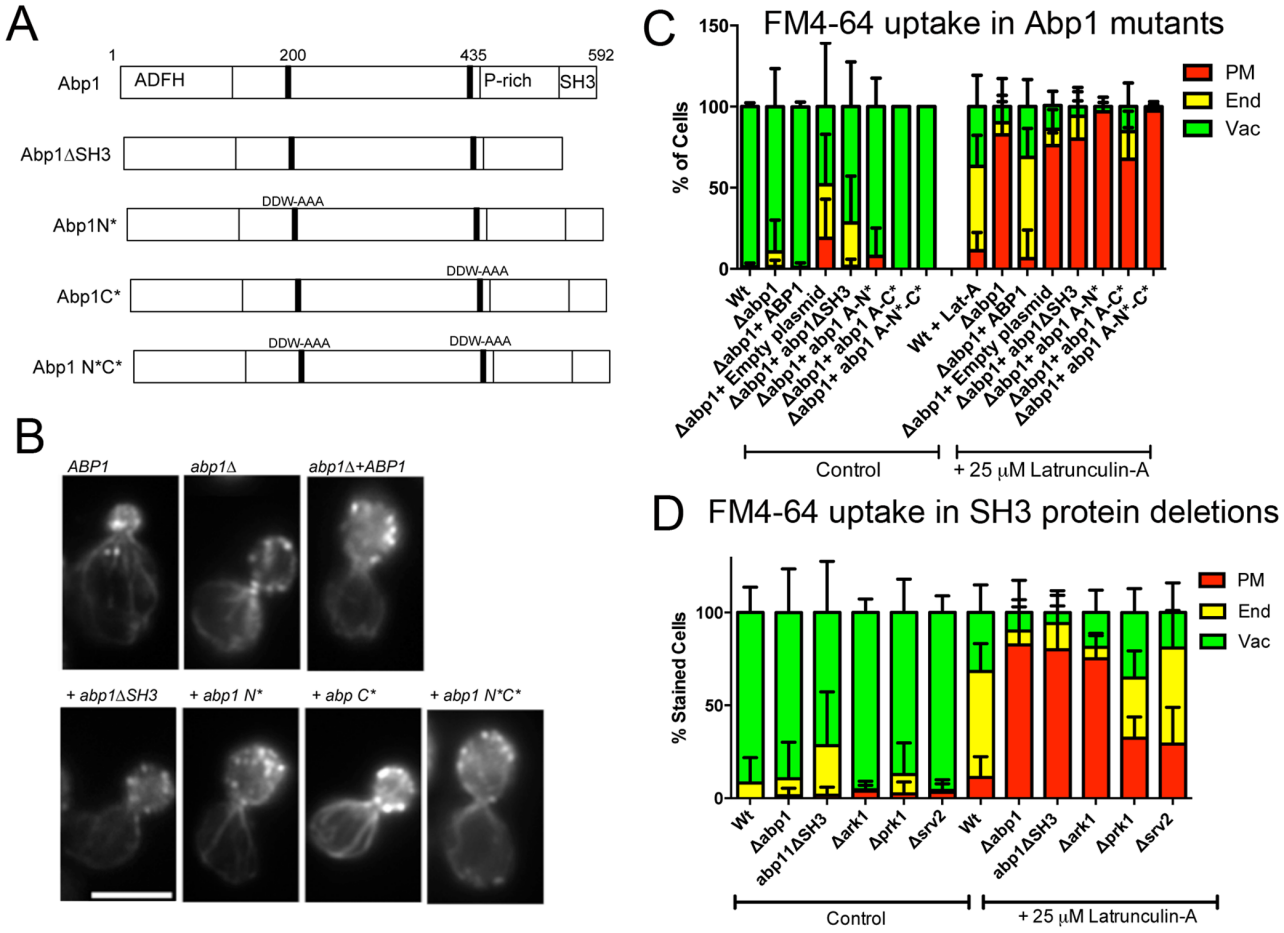
The Effect of Abp1 mutations on Bulk Endocytosis. (A) Schematic of full length Abp1 and the different mutants analysed in this study. (B) Wild type, *abp1*Δ and *abp1Δ* cells expressing wild type *ABP1* or mutant versions were grown to mid log phase before treating with Lat-A and following uptake of FM4-64. Cells were classified according to the most prominent staining after 20 minutes FM4-64 incubation. Error bars are standard deviation. (C) The effect of Abp1 mutations on actin organization as assessed by rhodamine phalloidin staining. (D) A number of proteins have been shown to bind to the SH3 domain of Abp1. Deletions in genes encoding 3 of these proteins, Ark1, Prk1 and Srv2/CAP were analysed to determine whether any of these interactions is potentially required for the Abp1 dependent endocytic pathway in the presence of 25 µM Lat-A. Cells were classified according to the most prominent staining after 20 minutes FM4-64 incubation. Error is Std Deviation.

To determine whether specific regions of Abp1 are required for its function in bulk endocytosis mutations were obtained or generated (Figure 5A). Abp1 has 2 acidic domains (N* and C*) previously characterized by Goode and colleagues as defective in Arp2/3 interaction but functional in actin binding. Three mutant forms of Abp1 (N*, C* and both N*C*) were kindly shared by D. Drubin (U.C. Berkeley) for this analysis [31]. In addition Abp1 has a C-terminal SH3 domain demonstrated to bind Ark1, Prk1, Scp1 and Srv2/CAP at endocytic sites [32], [33]. An Abp1 truncation lacking the SH3 domain was also generated. Cells lacking *abp1* were transformed with a plasmid carrying wild type or mutant *abp1*. *ABP1* was expressed in these cells under its own promoter on a centromere bearing plasmid. The effect of Abp1 mutants on actin organization was first analysed by rhodamine phalloidin staining to determine whether any defects could be visualized as a result of these mutations (Figure 5B). In all cases cells appeared to have relatively wild type characteristics and organization of both actin patches and cables. The effect of the Abp1 mutants on FM4-64 uptake in the presence or absence of 25 µM Lat-A was then analysed (Figure 5C). As expected in the absence of Lat-A most cells are able to endocytose FM4-64 at a similar level to wild type cells. The slight reduction in endosome to vacuole trafficking in the presence of the *abpl1ΔSH3* mutant suggests that this mutation is causing a dominant effect at a stage beyond initial endocytosis. In the presence of Lat-A, only wild type and *abp1* null cells transformed with *ABP1* showed clear FM4-64 uptake. Two mutants caused a very severe inhibition of uptake, these were Abp1 N* and Abp1 N*C* indicating the importance of the N terminal acidic site and the function of Arp2/3 for this endocytic route. The SH3 domain truncation also showed a severe defect, similar to the complete deletion supporting a role for SH3 binding interactions for Abp1 function in this endocytic pathway.

Given the importance of the SH3 domain of Abp1 in its function in this endocytic pathway, we considered whether known SH3 binding partners of Abp1 were important in this endocytic pathway [32], [33], [34]. Cells carrying deletions of the kinases *ark1* and *prk1* and of the cyclase activated protein, Srv2 were analysed for their effect on FM4-64 uptake. As shown in figure 5D only the deletion of *ark1* phenocopied the Abp1 SH3 truncation strongly suggesting that this kinase, but not its close homologue Prk1, functions in the Abp1 mediated endocytic pathway.

In order to understand the Abp1 dependent uptake pathway in more detail we sought to analyse behaviour of Abp1 under the low level Lat-A condition. Two strains were obtained expressing either Abp1-GFP or Abp1-mRFP with the tag integrated in the genome to generate Abp1 with a 7x alanine linker and the fluorescent tag at the C-terminus [9]. The lifetime of Abp1 at plasma membrane puncta was measured and small differences were observed. In particular the C-terminal GFP tag had a slightly longer lifetime in patches (Abp1-GFP 22.2±1 sec; Abp1-RFP 17.8±0.5 sec lifetime; error is SEM with n≥30 puncta from ≥9 cells). The proportion of puncta showing invagination was similar in both cases (Abp1-GFP 71±5%; Abp1-RFP 74±5% invagination).

**Figure 6:**
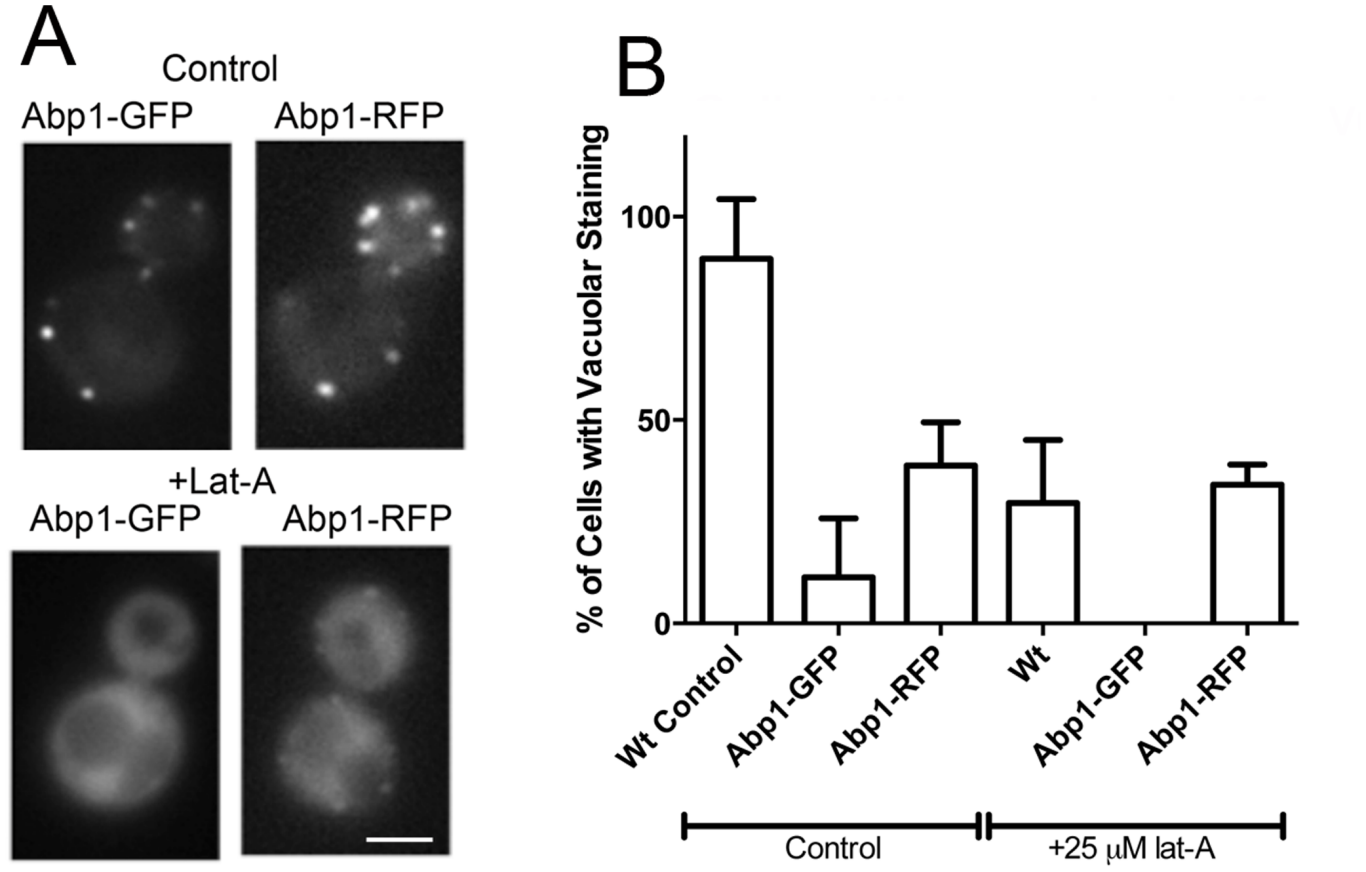
The effect of fluorescent protein tagging endocytic proteins in the presence and absence of Lat-A. (A) Abp1 with a C-terminal GFP and RFP tag was analysed in the presence of Lat-A. As shown, there is a marked reduction in signal in both cases, though some puncta can still be seen in the case of Abp1-RFP. (B) The effect of the tag on fluid phase uptake of Lucifer yellow was analysed in the presence and absence of Lat-A. The tag inhibits ability of the cells to endocytose LY in the presence and absence of Lat-A. Error is standard deviation. Bar = 2 µm.

The effect of 25 µM Lat-A was then analysed but unexpectedly the drug caused a marked loss in the localization of both tagged versions of the protein such that following Lat-A addition only 8±3% cells with Abp1-GFP and 71±6% cells with Abp1-mRFP showed localization in puncta. Even in the Abp1-mRFP tagged cells, while patches were visible they were diffuse and difficult to discern above background, compared to the fluorescence signal in the untreated cells. This very weak signal meant that analysis of movement of the patch has not been possible (Figure 6A). To determine whether the tag itself was potentially causing a defect in Abp1 function, the uptake of Lucifer yellow was analysed in cells expressing the C-terminally tagged Abp1 proteins. As shown (Figure 6B) both tagged proteins caused a defect in fluid phase uptake in the control cells indicating a dominant effect of the tag on normal endocytic function. In the presence of Lat-A, wild type cells showed reduced trafficking with about 20% of cells observed to traffic Lucifer yellow to the vacuole, the rest of the cells having endosomal staining. This level of uptake was mirrored in the Abp1-mRFP tagged cells, but not in the cells carrying Abp1-GFP indicating that the mRFP tagged Abp1 is able to function within this endocytic pathway despite not showing strong localization to endocytic punctae.

## Discussion

In this work we have investigated the uptake of bulk fluid and lipid which is able to enter cells even when the classical endocytic pathway is inhibited. Analysis of FM4-64 uptake in cells also expressing Sla1-GFP in wild type cells, revealed that this uptake can take place at distinct sites from one another. In addition, in budded cells FM4-64 can be observed to internalize in the mother and bud of cells while Sla1-GFP localizes mostly in the bud. FM4-64 uptake appears more diffuse than the puncta observed for the endocytic reporters such as Sla1-GFP (Supplementary movie), though the reason for this is not yet clear. Given that FM4-64 is found only in the endomembrane system and not in other membrane trafficking compartments this indicates that entry is likely to be mediated through some kind of vesicular carrier which then fuses with endosomes. It is important to note that even this uptake is inhibited with high levels of Lat-A indicating that it is still an actin mediated process.

Addition of low levels of Lat-A was shown to disrupt the normal CME route of endocytosis but not to cause disassembly of cortical F-actin structures nor to inhibit bulk endocytic uptake of fluid or lipid judged by uptake of the dyes Lucifer yellow or FM4-64. Under these conditions, both the endocytic reporter Sla1-GFP and the cargo GFP-Snc1 were inhibited in uptake. Analysis of proteins required when the classical route of endocytosis was inhibited reveal an Abp1-dependent endocytic pathway. This function of Abp1 requires both its SH3 domain and its acidic regions (N* and C*) which have been reported to interact with Arp2/3 [31]. Interestingly, a deletion of the gene encoding the kinase Ark1 but not the related Prk1, phenocopies the *abp1* deletion indicating a distinct/non-overlapping function for the Ark1 kinase. To date most work has focussed on Prk1, and the two proteins are often considered to be redundant kinases functioning in the same pathway [32], [35]. The result shown here indicates that while both kinases can interact with Abp1, their mode of function, or their relevant substrates, may only be available at some endocytic sites.

The question then arises as to what function Abp1 is performing at the endocytic sites. Over the years a number of functions have been ascribed to Abp1. *Abp1* deletion in yeast cells causes a reduced invagination rate of endocytic sites, while overexpression of *ABP1* is lethal [14]. Biochemically an interaction with Aim3 has been shown to generate an actin capping function, while a function in Arp2/3 activation has also been described [31], [36]. Its SH3 domain has been shown to be important for localizing Ark1 and Prk1 to endocytic sites, though interestingly, lack of kinase localization per se does not causes a very severe phenotype on analysis of endocytic reporter behaviour [32]. The data presented here suggests the possibility that Abp1 may function in a distinct, but overlapping endocytic pathway, that becomes the major internalization route, when the classic CME pathway is inhibited. This pathways does not appear to require the dynamin like protein Vps1 or the Arf3 GAP Lsb5. Another possibility is that residual function of the classical CME pathway is responsible for the uptake of FM4-64 and Lucifer yellow that is observed. If there was a such a residual function, it might be expected that occasional invagination of Sla1-GFP would be detected, and that GFP-Snc1 would be observed in some cell compartments. This however is not the situation detected in these experiments. In addition, the level of uptake of FM4-64 appears largely unimpaired which is difficult to correlate with CME which is functioning at a basal level. Thus, we consider that the Abp1-dependent uptake route is unlikely to be simply poorly functioning CME.

The actin requirement is likely to be Arp2/3-based as cortical actin patches are still observed in cells and because cells expressing the Abp1 N* and C* mutants, which are inhibited in Arp2/3 binding, are disrupted in the pathway.

Given the absence of actin cables in the treated cells, it would also seem less likely that Abp1 is functioning within the formin–based alternative pathway as formins are generally considered to function in cable production. However, it remains a possibility that the increased actin dynamics precludes normal formin function in cable generation and allows these proteins to function in a distinct role in endocytosis.

The alternative endocytic route identified here also shows a partial requirement for Sac6 and Rvs167. Sac6 is the yeast fimbrin homologue and is an actin bundling protein. Its ability to bind actin is necessary for invagination to occur [37], [38], [39]. The bundling of filaments is considered to make a stronger structure to allow invagination to occur against the effect of turgor pressure [30]. The reduction of FM4-64 and Lucifer yellow uptake in the absence of Sac6 supports the idea that actin is still important for this endocytic pathway. The level of Lat-A added is likely to have an impact on F-actin stability, as it is known that low levels of the drug are remedial for cells with stabilized actin structures [24]. It is not yet known whether these changes in stability impact on the subset of actin binding proteins that are able to interact with actin in these cells which in turn might affect the form of invagination that can be generated. A function for the amphiphysin Rvs167 in the Abp1-mediated pathway might provide a route for membrane curvature and possibly membrane scission. The yeast amphiphysins Rvs167 and Rvs161 are generally considered to function as an obligate dimer which can bind to phosphoinositol 4,5 bisphosphate containing membranes and generate curvature and possibly participate in lipid phase separation which might in some cases be able to lead to scission [40], [41].

Another outcome from this work is the demonstration of the negative effects of tagging. Much work in yeast has been generated from such studies but it is increasingly clear that while a tag might not be detrimental for growth in normal lab conditions, such tags are likely to affect at least a subset of protein function. In this case, different tags caused distinct levels of inhibition in Abp1 function supporting the importance of data from other approaches in drawing conclusions.

In summary, we have reported the existence of an Abp1- mediated endocytic pathway in *S.cerevisae*. The pathway continues to function in the presence of low levels of the actin monomer binding drug Lat-A, though F-actin is required for pathway function. We propose that yeast cells maintain distinct but overlapping endocytic pathways to allow fluid and lipid uptake even when environmental conditions may alter their capacity for more cargo driven routes.

## Supporting Information

Movie S1 Cells co-expressing Sla1-mRFP and Sla2-GFP (KAY1734) were imaged and composition of spots was analysed. Exposure 0.5 sec for each fluorophore. Time lapse of recording 1 frame/2 seconds. Movie shows at 1 frame/0.2 seconds.
(MOV)

Movie S2 Cells expressing Sla1-GFP (KAY733) were incubated with FM4-64FX for 5 minutes and imaged. Exposure 0.5 sec for each fluorophore. Time lapse of recording 1 frame/2 seconds. Movie shows at 1 frame/0.2 seconds.
(MOV)
